# Extended malaria parasite clearance time in African children following artemisinin-combination therapy enhances transmission *to Anopheles* mosquitoes

**DOI:** 10.1186/1475-2875-11-S1-O20

**Published:** 2012-10-15

**Authors:** Khalid B Beshir, Patrick Sawa, Chris J Drakeley, Amrish Y Baidjoe, Collins K Mweresa, Rahma U Yussuf, Sabah A Omar, Cornelus C Hermsen, Seif A Shekalaghe, Henk DFH Schallig, Robert W Sauerwein, Colin J Sutherland, Rachel L Hallett, Teun Bousema

**Affiliations:** 1 Department of Infection & Immunity, London School of Hygiene and Tropical Medicine, London, UK; 2 Human Health Division, International Centre for Insect physiology and Ecology, Mbita Point, Kenya; 3 Department of Medical Microbiology, Radboud University Nijmegen Medical Centre, Nijmegen, The Netherlands; 4 Kenya Medical Research Institute, Nairobi, Kenya; 5 Kilimanjaro Clinical Medical Research Institute, Kilimanjaro Christian Medical Centre, Moshi, Tanzania; 6 Ifakara Health Institute, Bagamoyo, Tanzania; 7 KIT Biomedical Research, Royal Tropical Institute, Amsterdam, The Netherlands

## Background

Artemisinin resistance was recently shown to have spread or emerged on the Thailand/Myanmar border. Evidence is accumulating that the parasite clearance time after artemisinin-based combination therapy (ACT) is increasing in settings in Asia and Africa. It is currently unknown if an extended parasite clearance time after ACTs has consequences for the individual patient or confers a higher malaria transmission potential.

## Methods and findings

298 children in Mbita, Western Kenya, with uncomplicated falciparum malaria were randomized to artemether-lumefantrine(AL, n = 153)ordihydroartemisinin-piperaquine(DP, n = 145). Parasite carriage post-treatment was determined by microscopy and qPCR, gametocyte carriage by quantitative nucleic acid sequence based amplication. Infectiousness to mosquitoes was determined by mosquito membrane feeding assays. Both drugs were efficacious as judged by standard trial outcomes. Sub-patent residual parasitaemia on day 3 was detected by qPCR in 36.11% (95% CI 25.11 - 48.29) of children treated with AL, and in 30.16% (95% CI 19.23 - 43.02) of children treated with DP. After adjustment for age, treatment arm and enrolment parasite density, children with an extended parasite clearance time were significantly more likely to have microscopically detected recurrent parasitaemia during follow-up (Odds Ratio: 19.51, 95% CI 5.24 - 72.71, p < 0.001). Children with an extended parasite clearance time were also more likely to be infectious to mosquitoes (Odds Ratio 2.76; 95% CI 1.14 - 6.67, p = 0.02) and gave rise to a higher oocyst load in mosquitoes (Incidence Rate Ratio 2.80, 95% CI 1.49 - 5.24, p = 0.001).

## Conclusions

Our findings indicate that an extended parasite clearance time after ACTs has consequences for the individual patient and for the population at large due to higher transmission potential. The high prevalence of residual sub-patent parasitaemia after treatment may be due to novel parasite genotypes with reduced drug sensitivity, inadequate population-level immunity, or the higher sensitivity of qPCR for detection of persisting parasites.

